# Using EMDR with autistic individuals: A Delphi survey with EMDR therapists

**DOI:** 10.1177/13623613221080254

**Published:** 2022-04-06

**Authors:** Naomi Fisher, Caroline van Diest, Marguerite Leoni, Debbie Spain

**Affiliations:** 1 Independent Practice, UK; 2 Synthesa Therapy, The Netherlands; 3 King’s College London, England

**Keywords:** autism, clinical supervision, EMDR, mental health, post-traumatic stress disorder, psychological therapy

## Abstract

Autistic individuals are at greater risk of experiencing adverse and traumatic life events. Eye Movement Desensitisation and Reprocessing (EMDR), a psychological therapy, is potentially effective for treating the constellation of difficulties arising from traumatic experiences, as well as mental health conditions. Yet minimal research has focused on how EMDR may require adaptation to improve its accessibility, acceptability and effectiveness for autistic individuals. In a three-round Delphi survey, 103 EMDR therapists were asked about barriers to EMDR for autistic individuals and adaptations employed to enhance therapy, so as to generate consensus about important or essential components of adaptations to EMDR. Four types of barriers were highlighted: client-related characteristics, therapist-related characteristics, differences in the therapeutic relationship and systemic issues. One hundred and twenty-four adaptations were identified, including 35 general adaptations (i.e. relevant across EMDR phases), 81 relating to specific EMDR phases and 8 about EMDR clinical supervision. Of these, 27 adaptations were used often or always by at least 80% of participants; a further 61 were sometimes incorporated within therapy, depending on the client. Study findings highlight the need for EMDR therapists to have training about autism and the potential ways of tailoring EMDR, and that individual case conceptualisation is key.

**Lay abstract:**

Eye Movement Desensitisation and Reprocessing (EMDR) is a psychological therapy that can help people process memories and distress about past events, so they have less impact on their daily lives. EMDR can be effective for treating symptoms of post-traumatic stress disorder, including nightmares and anxiety. Psychological therapies usually require adaptation so they are more accessible and effective for autistic people, but minimal research has focused on how best EMDR can be adapted. In this online survey study, we asked 103 EMDR therapists about barriers they think autistic people face when trying to have EMDR and what adaptations they use in their everyday practice. Four barriers were highlighted: client-related characteristics, therapist-related characteristics, differences in the therapeutic relationship and broader issues. Therapists identified a range of adaptations that can potentially be useful for autistic people, relating to being flexible, communicating clearly and having an awareness of individual differences. Many therapists emphasised the importance of not making assumptions about a person based on their autism diagnosis. Overall, the study findings suggest adaptations to EMDR are likely to be useful, but how relevant they are depends on each person.

## Introduction

Autism spectrum disorder (henceforth, autism) is a childhood onset neurodevelopmental condition. Autism is characterised by (1) differences (impairments) in social communication and interaction and (2) engagement in restricted or repetitive patterns of behaviour, interests or activities (American Psychiatric Association (APA), 2013). The severity and impact of core autism traits varies between individuals (APA, 2013). Some individuals present with marked traits that are noticed early in childhood; others present with subtle traits that are only diagnosed in adulthood. Males are diagnosed more frequently than females, with a reported gender ratio of 3:1 (Loomes et al., 2017).

Autistic individuals experience disproportionately high rates of mental health conditions, including anxiety and affective disorders, eating disorders, psychosis and post-traumatic stress disorder (PTSD) (for review, see Hossain et al., 2020). Complexities in assessing mental health, for example, due to inaccessible services, poor clinician understanding of autism and diagnostic overshadowing, mean comorbidities are often undetected or misdiagnosed. Mental health comorbidity can negatively impact daily functioning, attainment of independence and quality of life. Development of accessible, acceptable and effective interventions to target comorbidities is, therefore, a priority.

### Autism and PTSD

There is increasing interest in understanding trauma and PTSD experienced by autistic individuals (Peterson et al., 2019; Rumball, 2019; Stack & Lucyshyn, 2019). PTSD can occur following directly or vicariously experiencing, witnessing or learning about single or multiple traumatic events, that involve ‘actual or threatened death, serious injury, or sexual violence’ (APA, 2013). PTSD symptoms include (1) re-experiencing traumatic events (e.g. through intrusive memories, flashbacks, nightmares); (2) avoiding or suppressing trauma-related memories and cues; (3) negative changes in mood (e.g. feelings of horror, anger, guilt) and cognition (e.g. negative beliefs about the self or others), difficulties recalling aspects of traumatic events and feelings of detachment; (4) hyperarousal (e.g. hypervigilance, exaggerated startle response, poor sleep); and (5) significant distress and functional impairment (APA, 2013).

PTSD prevalence in clinical samples of autistic individuals is estimated to be between 2% and 17%, compared to 3% in neurotypical individuals (Rumball, 2019). However, clinicians do not always screen for, and treat, trauma-related symptoms in autistic individuals (Kerns et al., 2020), so this may be an underestimate. Several risk factors increase vulnerability for PTSD development in autistic individuals (Peterson et al., 2019). These include increased exposure to traumatic events (e.g. victimisation, maltreatment, bullying), and regularly experiencing events that constitute ‘small traumas’ (i.e. events that are traumatic for the individual, but do not meet stringent diagnostic definitions of a traumatic event; Shapiro, 2018). It is also possible that, for some autistic individuals, there is an atypicality (or potentially, an autism-specificity) in terms of events construed as traumatic (Kerns et al., 2015; Rumball et al., 2020), for example, relating to changes to routines, rule-breaking of others, difficulties in making sense of events and sensory processing difficulties.

### Evidence-based psychological therapies for PTSD

Psychological therapies are the treatment of choice for PTSD in neurotypical individuals, with strong empirical support for Eye Movement Desensitisation and Reprocessing (EMDR; Lewis et al., 2020; National Institute for Health and Care Excellence (NICE), 2018).

EMDR was developed by Francine Shapiro in the 1980s as a therapy for PTSD (Shapiro, 1989). Shapiro proposes that all human beings have the capacity to heal naturally, physically and psychologically, conceptualised as an adaptive information processing model (AIP). However, if adverse events occur and individuals cannot naturally heal, EMDR can facilitate the process. The theoretical basis for EMDR is that adverse experiences are stored unprocessed in the brain in a sensory form (with images, thoughts, physical sensations and/or emotions). Similar to Brewin’s Dual Representation Model (somatic and narrative memory) (Brewin et al., 1996), this affects the individual in the present day (physically, emotionally and behaviourally). EMDR requires the pairing of an emotionally activated memory with alternative bilateral stimulation (BLS; e.g. eye movements, tapping, auditory sounds). This facilitates connection to the AIP and enables the memory to be processed, thus relieving the influence and impact on daily life and functioning (Shapiro, 1989).

EMDR is an eight-phased treatment: Phase 1 involves history taking; Phase 2 prepares for emotional regulation during processing; Phases 3–7 focus on active processing of memories, desensitising distress and reconsolidating memories; and Phase 8 entails re-evaluating the memories processed, the clients’ presentation or symptoms and achievement of therapy goals. The optimal number of EMDR sessions varies. Some individuals benefit from three sessions to process a single traumatic event, whereas others require substantially more. There is also growing evidence that EMDR can be effective for conditions other than PTSD, including mood disorders, substance misuse, panic disorder and unexplained symptoms (for review, see Sepehry et al., 2021; Valiente-Gómez et al., 2017; van Rood & de Roos, 2009).

### EMDR for autistic individuals

EMDR may be particularly suited for autistic individuals for several reasons (Fisher & van Diest, 2022). EMDR is client-led, adapting well to different levels of cognitive ability. Memories do not need to be put into words, making it relevant for those whose verbal language may be less well developed or who struggle to interpret their experiences or internal worlds. When used by experienced therapists, EMDR can be highly flexible and responsive to individual differences. EMDR can be facilitated by visualisation, emotional experience, somatic sensation or thought. EMDR can also be used for processing upsetting situations in the present or those anticipated in the future that are associated with distress, anger, confusion or fear. This makes it accessible for individuals who may not easily make the connection between past experiences and present-day distress (Shapiro, 2018).

To our knowledge, only five studies have examined the feasibility and/or effectiveness of EMDR for autistic individuals: one case study of a 21-year-old woman diagnosed with Asperger syndrome, receiving EMDR for PTSD symptoms (Kosatka & Ona, 2014), three case series describing EMDR for PTSD symptoms in up to six individuals with an intellectual disability, at least one of whom had a concurrent autism diagnosis (Barol & Seubert, 2010; Mevissen et al., 2011, 2012), and one randomised controlled trial (RCT) of EMDR for 21 autistic adults diagnosed with autism and no concurrent intellectual disability, presenting with clinically significant PTSD symptoms or a PTSD diagnosis (Lobregt-van et al., 2019).

Studies share some commonalities. Other than one study that offered three sessions per week for the first 8 weeks (Kosatka & Ona, 2014), clinicians delivered Shapiro’s eight-phase protocol or the very similar Dutch variation of this, generally weekly, for up to 17 sessions. Adaptations were incorporated into EMDR to make this more suited to the preferences and needs of autistic individuals and/or individuals with an intellectual disability. These included (1) spending longer developing rapport, taking a history and on the preparatory stabilisation phase; (2) opting for alternative BLS to eye movements (e.g. hand buzzers, headphones, audio speakers); (3) using the storytelling method; (4) using a child, rather than adult treatment protocol; (5) reducing emphasis on identifying negative and positive cognitions; (6) therapists being more directive during processing; (7) writing information down; (8) offering options rather than open-ended questions (e.g. suggesting words to describe emotions/feelings for individuals with reduced emotional literacy); (9) varying the session duration; and (10) another person being present in sessions (e.g. a parent). Across studies, PTSD symptoms were reported to have improved post-intervention, evident by a change in Subjective Units of Distress (SUDS) or Validity of Cognition (VoC) ratings, or on standardised self-report measures.

### Study rationale and aims

In sum, empirical and clinical findings indicate that autistic individuals experience PTSD, potentially at higher rates than neurotypical individuals. As with interventions for other mental health comorbidities, very few studies have focused on feasibility, acceptability and/or effectiveness of psychological therapies to treat PTSD in autistic individuals (Rumball, 2019). EMDR may be a particularly suitable treatment option for reasons outlined above, subject to appropriate adaptations to accommodate autistic individuals’ preferences and needs. As yet there has been no systematic research focusing on what these should comprise, and why. While prior, but limited, research has investigated adaptations to other psychological therapies for this client group (e.g. cognitive behaviour therapy (CBT); Kerns et al., 2016; Spain & Happé, 2020), there may be issues with directly extrapolating findings, given some of the distinct aspects of EMDR as an approach.

This study aimed to develop therapist consensus about (1) adaptations to EMDR that are important when working with autistic individuals, so that this is more accessible, acceptable and effective and (2) autism-relevant considerations for EMDR clinical supervision.

## Methods

### Study design

We employed Delphi survey methodology, an iterative study design useful for developing consensus in cohorts of individuals (e.g. practitioners) with experience/expertise about under-examined topics (Langlands et al., 2008). The online survey comprised three rounds.

### Ethical approvals

Ethical approvals were obtained (REC reference MRA-20/21-21063, King’s College London). Participants gave informed consent (via the online survey) and were informed the study would be written up for dissemination purposes. The article contains no person identifiable information.

### Participants

We used convenience sampling methods and recruited via inter/national clinical academic networks, special interest groups, EMDR associations and social media. Study inclusion criteria were (1) internationally based therapists working in any setting, with any clinical population; (2) with at least some formal EMDR training; (3) limited through to advanced proficiency in EMDR; (4) working with autistic individuals, of any age, occasionally, sometimes or often; and (5) using English sufficiently proficiently to complete the survey.

One hundred and three participants completed the Round 1 survey. See Table 1. This included medical doctors, psychiatrists, chartered psychologists (including clinical, counselling, occupational, forensic and educational psychologists), neuropsychologists, CBT therapists, psychotherapists (including drama, art and music psychotherapists), counsellors, nurses, social workers and occupational therapists. Numerous participants held several professional qualifications. Eighty-three participants worked in the United Kingdom, five in Australia, four in the United States, one in Egypt, one in Greece, one in the Netherlands and one in New Zealand.

**Table 1.** Participant demographics.

	Round 1 (n = 103)	Round 2 (n = 43)	Round 3 (n = 26)
Population worked with			
Children and adolescents	6	1	2
Adults	31	18	10
Lifespan	61	24	14
Level of EMDR training			
Not yet finished basic training	1	1	1
Basic adult training	57	26	16
Basic child training	27	6	6
EMDR practitioner	26	8	4
EMDR consultant	20	7	3
EMDR training facilitator/trainer	4	1	-
Years of experience of providing EMDR			
1–4	47		
5–9	22		
10–14	17		
15–20	9		
20+ (range: 1–32)	2		
Experience of working with autistic individuals			
Occasionally	28		
Sometimes	17		
Regularly	47		
All the time	6		
Experience of working with individuals with an intellectual disability	42		

EMDR: Eye Movement Desensitisation and Reprocessing.

Participants worked in child and adult psychological therapies services, community mental health services, forensic settings, independent practice, education, tertiary services, the military, voluntary organisations and services for individuals with an intellectual disability.

Specific information about participants’ personal characteristics (e.g. age, gender, socio-economic status) was not sought, as this was not relevant to the study aims.

### Survey development

#### Round 1 survey

The initial survey comprised three sections, focusing on

1. General demographic information (e.g. core profession, work setting and experience, relevant training and experience of delivering EMDR);
2. Perceived barriers to EMDR accessibility and/or effectiveness for autistic individuals, and adaptations employed to accommodate these in each therapy phase;
3. Additional autism-specific considerations for clinical supervisees/supervisors, when using EMDR.

#### Round 2 survey

The Round 2 survey comprised 124 statements generated by thematically analysing responses to Round 1. These statements included

- Thirty-five general adaptations (i.e. relevant across EMDR phases);
- Eighty-one adaptations relating to specific EMDR phases;
- Eight adaptations about EMDR clinical supervision.

#### Round 3 survey

The Round 3 survey contained 10 statements about adaptations to standard EMDR and 1 statement about clinical supervision that had not attained consensus in Round 2. We also included an open question to clarify reasons why participants had rated statements, ‘I sometimes do this, depending on the client’ in Round 2.

Rounds 2 and 3 repeated some demographic questions from Round 1 to contextualise findings.

### Measurement scale

Following similar Delphi survey measurement scales cited elsewhere (e.g. Langlands et al., 2008; Morrison & Barratt, 2010; Spain & Happé, 2020), participants rated the degree to which they agreed with statements in Rounds 2 and 3 (see Table 2).

**Table 2.** Measurement scale for survey statements in Rounds 2 and 3.

Adaptations to EMDR	Adaptations to clinical supervision
1. I always do this	1. This is essential for all
2. I often do this	2. This is important
3. I sometimes do this, depending on the client	3. This could be useful, depending on the client group
4. I never do this	4. This is not important
5. I think this should not be done at all	5. This is actively unhelpful

EMDR: Eye Movement Desensitisation and Reprocessing.

### Procedure

The study took place between November 2020 and April 2021. Participants accessed the surveys via Qualtrics (https://www.qualtrics.com/uk/). Surveys were formatted similarly, with demographic questions initially, followed by more specific questions (Round 1), or statements (Rounds 2 and 3). There were also options for comments and free text.

### Community involvement

We sought informal feedback about the scope and aims of the first survey, categories of questions included and barriers to EMDR from one autistic adult. We informally discussed the study findings and interpretation with two autistic adults and a parent/carer of an autistic child. Participants included autistic therapists.

### Data analysis

Data were analysed using mixed methods. Demographic data were analysed descriptively. Participants’ free-text comments in Round 1 were analysed thematically (Braun & Clarke, 2006); comments were exported into a Word transcript and reviewed by D.S. and M.L. Data were coded and clustered into one of the three overarching themes: (1) general adaptations to EMDR; (2) EMDR phase-specific adaptations; and (3) adaptations pertaining to clinical supervision. When participants’ suggestions related to at least one theme (e.g. avoiding using metaphor, assessing and accommodating sensory sensitivities and preferences, embedding flexibility and creativity), these were incorporated into the general adaptations category.

For Rounds 2 and 3, we established the proportion of participants rating each statement similarly. Consensus was defined as statements rated 1 or 2 on the Likert-type scale by ⩾80% of participants. Statements rated 1 or 2 by 70%–79% participants in Round 2 were re-rated in Round 3. Statements attaining consensus lower than 70% agreement were excluded. We calculated percentages rounded up to a whole number, due to changes in the number of participants per survey.

## Results

### Round 1

#### Rationale for using EMDR

Participants reported they used EMDR as an intervention for numerous conditions, including trauma, PTSD and adjustment to adverse life events, as well as phobias, anxiety disorders, obsessive compulsive disorder (OCD), depression, addictions, adjustment disorders, nightmares, eating disorders, symptoms of attention deficit hyperactivity disorder (ADHD) and behaviours associated with personality disorders.

#### Barriers to accessible and effective EMDR

Participants identified four categories of potential barriers to the accessibility and effectiveness of EMDR for autistic individuals:

1. Client characteristics, for example, differences in communication, difficulties with emotion regulation, beliefs about self and others which made it hard for clients to process effectively and high anxiety about trying something new.
2. Therapist characteristics, for example, a lack of confidence in working with this client group, difficulties in sitting with uncertainty and limited clarity about how to stay on track.
3. Differences in the therapeutic relationship, for example, therapists reported finding it difficult to know whether an adequate therapeutic relationship had been established with this client group, meaning they could not easily judge whether they could proceed safely with EMDR.
4. Systemic issues, for example, negative narratives about autism and a lack of understanding in the people around a client.

#### Adapting EMDR for autistic individuals

Participants identified numerous adaptations they employed to address or accommodate barriers. These were synthesised into statements for Round 2 (see Tables 3 and 4, and Supplemental Information Tables 1 to 4).

**Table 3.** Elements of EMDR that ⩾80% of therapists often or always incorporate in therapy with autistic clients.

	% of participants endorsing Items 1–2
*General adaptations to EMDR*	
Normalise experiences	88
Use very clear language. Do not assume that they have necessarily understood what you intended to say	86
Be flexible and creative	91
Be aware of how they communicate their level of arousal through behaviour and use this information to evaluate how they are coping during sessions	84
Take time to understand the language they use around thoughts and emotions, and mirror this	91
Respond to the person in front of you. Some may take well to rating scales and questions about cognitions, others may not at all	91
Be open to learning from the client and celebrate each person’s uniqueness	95
Consider the role of autism within the conceptualisation	86
*Phase 1: History taking*	
Consider small ‘t’s as well as big ‘T’s as possible targets	98
*Phase 2: Preparation stage*	
Use their background and history to identify resource possibilities	88
Try a range of different types of BLS	84
*Phase 3: Assessment phase*	
General	
Be particularly mindful of language in this phase as people may be very sensitive to failure and ‘getting it wrong’	80
Emotions	
Use their own language to describe emotions	83
*Phase 8: Re-evaluation*	
Make time for the person to debrief about their week	80

BLS: bilateral stimulation; EMDR: Eye Movement Desensitisation and Reprocessing.

**Table 4.** Aspects of EMDR clinical supervision that ⩾80% of therapists deemed important or essential when working with autistic clients.

	% of participants endorsing Items 1–2
Supervisor should have knowledge about autism and trauma	88
Supervisor should have knowledge of other neurodevelopmental conditions such as ADHD and dyslexia	80
Supervisor should have knowledge of attachment theories	95
The supervisory relationship should be one within which admitting to going off-piste is possible	93
There should be time to discuss issues to do with the therapeutic relationship and how autism might affect this	93
There should be time to discuss different ways of approaching therapy, how to adapt this and how to overcome roadblocks	98

EMDR: Eye Movement Desensitisation and Reprocessing; ADHD: attention deficit hyperactivity disorder.

### Round 2

Forty-three participants participated in Round 2.

This survey comprised 124 statements. Of these, 14 were rated by at least 80% of participants as adaptations that they always or often included in EMDR with autistic individuals (see Table 3). In addition, at least 80% of participants rated 6 considerations relating to EMDR clinical supervision as aspects that are essential or important when working with autistic individuals (see Table 4).

Twelve Round 2 statements required re-rating in the Round 3 survey (marked with an asterisk in Supplemental Information Tables 1 to 3), as these attained consensus from 70% to 79% of participants.

We noted that many participants rated several Round 2 statements, ‘I sometimes do this, depending on the client’. To explore this further, we included an additional question in the third survey, to better understand this (see Table 5).

**Table 5.** Factors accounting for statements being rated ‘sometimes / it depends’ in Rounds 2 and 3.

	Number of times endorsed by participants
Client’s clinical presentation (e.g. complex comorbidity)	18
Client’s cognitive capacity (e.g. diagnosed with a learning disability or has acquired brain injury)	12
Based on previous clinical experience (e.g. of working with clients with similar presentations)	12
Quality of therapeutic alliance with client	11
Confidence in deviating from standard EMDR protocols	8
Risk (e.g. concerns about self-harm or suicidality)	7
Age of client group (e.g. young children)	6
Amount of experience of using a range of EMDR protocols	5
Amount of experience of working with autistic people	4
Quality of clinical supervision (e.g. clinical supervisor has limited experience or expertise in working with autistic people)	4
Up-to-date knowledge of the research evidence base (e.g. incorporating new research findings into clinical practice)	4
Preferences for using particular EMDR protocols	2
Service constraints (e.g. only allowed to provide a specified number of sessions)	1

EMDR: Eye Movement Desensitisation and Reprocessing.

### Round 3

Twenty-six participants completed the third survey.

The Round 3 survey included 12 statements to be re-rated, 11 relating to EMDR and 1 pertaining to clinical supervision. Overall, a further seven statements pertaining to adaptations to EMDR attained consensus scores of 80%.

Across Rounds 2 and 3, participants consistently endorsed some statements as ‘I sometimes do this, depending on the client’. We therefore also extracted statements that attained this rating by at least 80% of participants (see Supplemental Information Tables 1 and 2). Table 5 outlines reasons why participants rated statements as such.

### Summary of results

Overall, 21 statements relating to adaptations were rated as often or always being incorporated into EMDR with autistic individuals, and 6 adaptations were deemed important or essential for EMDR clinical supervision. A further 57 statements were rated as sometimes being incorporated into EMDR therapy and 3 statements about clinical supervision were considered useful (see Supplemental Information Tables 1 and 2). Thirty-seven statements did not attain consensus ratings above 69% (see Supplemental Information Table 3). No statements were rated ‘this should not be done’.

## Discussion

Emerging research suggests EMDR may be beneficial for autistic individuals, and much anecdotal evidence indicates this is being used clinically, often without much guidance. EMDR therapists describe finding their own way, in the absence of autism-specific resources and training. This study focused on therapists’ perspectives about barriers to EMDR for autistic individuals, development of consensus about adaptations that are important for EMDR when used with autistic individuals and autism-relevant considerations for EMDR clinical supervision.

Participants identified four principal barriers that can impede access to, or effectiveness of, EMDR for autistic individuals, including those that are client- and therapist-related, as well as differences in the therapeutic relationship and systemic issues. No other studies, to our knowledge, have specifically examined barriers to EMDR for this client group. However, in a recent systematic review, Adams and Young (2021) synthesised data from 12 studies examining barriers and facilitators to psychological therapy (predominantly CBT) for autistic individuals. Importantly, studies reviewed incorporated data obtained from multiple stakeholders, including autistic individuals and families. There is some overlap between the review findings and barriers found in this study, such as therapists’ limited awareness of how to adapt interventions and clients’ communication difficulties.

Broader barriers were also highlighted in the review, including those that are service-related (e.g. lack of services, long waiting times) and problems with services/therapists failing to recognise severity of symptoms in autistic individuals, and thus, the need for formal intervention (Adams & Young, 2021). These latter points may be especially salient in the context of EMDR, as evidence suggests health professionals may not routinely ask autistic individuals about trauma-related symptoms (Kerns et al., 2020), and the nature of events construed as traumatic may be idiosyncratic (Peterson et al., 2019). Conceivably, in practice, this means some autistic individuals may not be offered EMDR even if this is potentially indicated as a treatment option. Clients should ideally have opportunities for informed discussions with therapists about which therapeutic approach may be a good fit for them (Stark et al., 2020), why (e.g. based on presenting problems, goals for intervention, the evidence base) and how to evaluate if this is the case. Taken together, there is an impetus to better understand the range of barriers that might affect access to and effectiveness of EMDR for autistic individuals from all stakeholders’ perspectives, so that the care pathway can be better tailored, and autistic individuals are offered a range of evidence-based therapies to address mental health symptoms (Harper et al., 2019).

Study findings show that little can be taken for granted when working with autistic individuals. We know there is substantial heterogeneity within this client group (Jeste & Geschwind, 2014), and this is reflected in our data. As one participant said, ‘no sweeping generalisations here’. Participants emphasised the importance of approaching each client as an individual.

EMDR processing follows a scripted ‘Standard Protocol’. This standard protocol is applied to a wide range of psychological problems. Slight variations on this exist for special situations, such as Pain or Phobias. Therapists sometimes ask for a scripted ‘Autism Protocol’, laying down exactly what should be said with this client group. No formally scripted autism protocols exist that we have been able to find, although we have found two unpublished documents (Lievegoed et al., 2013; Paulson, 2003/personal communication), that outline guidelines and adaptations for working with autistic individuals.

Findings in this study suggest that developing a Toolbox or conceptual framework for working with autistic individuals may be useful, emphasising flexibility and adapting to the individual client. Encouraging therapists to respond in this way, rather than to their diagnosis, may be particularly important for this client group, as evidence suggests that professional bias about autism and autistic individuals can impact negatively on service provision (Como et al., 2020; Fennell & Johnson, 2021).

Overall, we found that the adaptations used by the greatest number of therapists (i.e. those that attained consensus of more than 80%) were general therapeutic points, rather than EMDR-specific. These core adaptations were broadly based on three principles: flexibility, clear communication and an awareness of individual differences. Prior Delphi survey research has reported similar conclusions, whereby there was greater consensus between therapists with autism-expertise about general adaptations to the structure and process of CBT for autistic individuals, rather than about very specific CBT interventions (Spain & Happé, 2020).

An interesting issue emerged in this study about the nature of consensus. EMDR therapists were asked to rate how often they might employ adaptations with autistic individuals, using a Likert-type scale. The middle rating ‘3’ was defined as ‘I sometimes do this, depending on the client’. In a typical Delphi survey, only a strong endorsement of an item would be used for the consensus rating (e.g. Langlands et al., 2008). For this study that would mean only ratings of ‘1’ and ‘2’ would be included (indicating that the therapist always or often does this). However, given the heterogenous nature of the client group, it was felt that important items might be excluded by only focusing on these ratings. For example, an adaptation could be very important for therapists to consider, but only when working with individuals who struggle to verbalise their feelings, or those with an intellectual disability. Equally, some therapists may be working mostly with autistic individuals who are able to access the standard EMDR protocol with few adaptations, and this could overshadow the responses of those who worked with individuals requiring more adaptations. We therefore also calculated the less conservative consensus ratings including ‘3’ (see Supplemental Information Tables 1 and 2). To investigate this issue further, participants were asked why they had rated statements this way (Table 5), and they described reasons including flexibility, adopting a client-focused approach, individualised case conceptualisation and when they used an adaptation sometimes, but not in every session.

This less conversative approach generated quite a different data set. Here, we found suggestions for more EMDR-specific adaptations. Participants suggested ways to make phases of the EMDR protocol more accessible, while still preserving the core purpose of each phase. Adaptations to history taking (Phase 1) included the need for building information over time with the support of others around the individual, gathering information with visual aids if possible and focusing upon an individual’s strengths. The preparation phase (Phase 2) included suggestions about presenting information visually (e.g. pictures, diagrams, props) and integrating special interests when resourcing (e.g. super hero figures, characters from history). Within the activation phase (Phase 3), participants endorsed using visual aids and being flexible in how they approached the positive and negative cognition, and identification of targets. During the densensitisation phase (Phase 4), adaptations included being both more directive and controlled (e.g. with more directive interweaves and shorter BLS sets), and also being more client-led (e.g. allowing clients to control the length of BLS sets). Again, these differences potentially reflect the highly varied nature of this client group. Adaptations incorporated in the last three phases (installation, closure and re-evaluation) were mostly general in nature, such as taking longer to close down sessions and doing more cognitive work if necessary. Many participants recommended ending with a relaxing and positive activity and focusing on quality of life between sessions.

It is possible that some adaptations may relate to attempts to limit emotional and sensory overload (e.g. titrating exposure to emotional memories), which fits with research on information processing and sensory processing difficulties experienced by some autistic individuals (Hazen et al., 2014; Mazefsky, 2015). The recommendation for additional time for closure and providing concrete information regarding expectations are consistent with this. Difficulties generalising was also put forward as a possible difference between using EMDR with autistic individuals as opposed to a non-autism population. Taken together, adaptations identified here are in keeping with, but more comprehensive, than those reported in the few EMDR intervention studies for autistic individuals (e.g. Barol & Seubert, 2010; Kosatka & Ona, 2014; Lobregt-van et al., 2019; Mevissen et al., 2011, 2012)

The final category of adaptations included those that fewer than 80% of participants said they used sometimes, often or always, such as using adjunctive techniques and more simplified strategies (e.g. a child protocol with adults). Given the heterogeneity of this client group, these adaptations may still be important for some. Also, in some instances, a low consensus rating may reflect the reality that not all the participants had attended extra training required to use these approaches (e.g. constrained by service models), rather than reflecting utility or appropriateness of the technique itself.

Finally, there was a high degree of consensus on considerations relating to clinical supervision, for example, that supervisors need to be knowledgeable about autism and associated (neurodevelopmental) conditions, and that the supervisory context should allow for appreciation of the relevance of autism within the case conceptualisation and therapeutic relationship. These findings lend support to the idea that improved understanding of autism may impact therapists’ capabilities and confidence to work with this client group (for review, see Corden et al., 2021; see also, Lipinski et al., In press; Maddox et al., 2019), highlighting the potential need for health professionals to be offered autism training as part of core training. In the United Kingdom, for example, the three-tiered Oliver McGowan Mandatory Training in Learning Disability and Autism is due to be rolled out to all public facing workers, and aims to equip professionals with sufficient knowledge and understanding about autism so that they can tailor style and interventions as needed. Very few studies have examined the impact of training for therapists, but there is tentative evidence in support of this (Corden et al., 2021).

### Study limitations

This study has several limitations. The nature of the recruitment strategy meant we could not monitor how many people saw the study advertised versus how many participated, or their motives for participation. Knowledge of autism and EMDR proficiency were not objectively assessed. Participants were not asked directly if they are autistic (at least three disclosed this), and the views of autistic individuals about EMDR were not garnered in this study. The sample was not large enough to analyse sub-group responses separately (e.g. according to whether participants worked with young people vs adults, or individuals with or without an intellectual disability). We were also unable to analyse sub-group data based on years of EMDR experience, or years of practice of using EMDR with autistic people. Together, this may have affected consensus-building on items more relevant for particular sub-groups. The sample size diminished each round – this is not uncommon in Delphi surveys but may affect generalisability of findings. Finally, the survey focused specifically on adaptations made in the therapy room. This of course supposes that autistic individuals had accessed the therapy room, something many find extremely challenging due to broader barriers.

## Conclusion

This study investigated, for the first time, EMDR therapists’ perspectives about barriers to EMDR for autistic individuals and sought to develop consensus about requisite adaptations to enhance treatment accessibility, acceptability and effectiveness. Overall, study findings indicate therapists are using EMDR with autistic individuals in a wide range of settings, and that they are enthusiastic about the possibilities EMDR offers, both as a treatment for PTSD, as well as other mental health conditions. The general pattern of the data indicated few specific adaptations to the EMDR protocol apply to all autistic individuals. Further, since autistic individuals are so heterogenous, the adaptations necessary are likely to be varied. It may be useful to think in terms of a ‘therapeutic toolbox’, with flexibility, clear communication and responsiveness to the individual as guiding principles. Clearly, knowledge about autism and adequate autism-informed clinical supervision are also deemed key for ensuring tailored EMDR practice can be offered. Further research is now needed to better understand autistic individuals’ perspectives about EMDR, as well as whether incorporating adaptations into therapy gleans more favourable outcomes. More research focusing on how EMDR therapists can acquire more knowledge, skills and confidence in relation to working with autistic individuals is also warranted.

## Supplemental Material

sj-docx-1-aut-10.1177_13623613221080254 – Supplemental material for Using EMDR with autistic individuals: A Delphi survey with EMDR therapists Click here for additional data file. Supplemental material, sj-docx-1-aut-10.1177_13623613221080254 for Using EMDR with autistic individuals: A Delphi survey with EMDR therapists by Naomi Fisher, Caroline van Diest, Marguerite Leoni and Debbie Spain in Autism
