# Supplementary material for: Using EMDR with autistic individuals: A Delphi survey with EMDR therapists
Source: Autism. 2022 Apr 6;27(1):43–53. doi: 10.1177/13623613221080254 (PMC9806468; doi:10.1177/13623613221080254)
Supplement: sj-docx-1-aut-10.1177_13623613221080254 – Supplemental material for Using EMDR with autistic individuals: A Delphi survey with EMDR therapists [file sj-docx-1-aut-10.1177_13623613221080254.docx]

Supplementary information

Table 1 Elements of EMDR that ≥ 80% of therapists sometimes incorporate in therapy with autistic clients

|  | % of participants endorsing items 1-3 |
| --- | --- |
| ***General adaptations to EMDR*** |  |
| Assess sensory preferences and sensitivities | 93 |
| Be more directive in style (i.e., less socratic, with fewer open-ended questions) | 100 |
| Use visual aids (e.g., drawing, pictures, videos) | 86 |
| Avoid metaphor | 93 |
| Ask about and include special interests throughout the therapy | 98 |
| Always offer sessions at the same time and place. | 100 |
| * Take a graduated/progressive approach towards full trauma processing | 100 |
| Use Flash | 81 |
| Focus on quality of life and functioning | 93 |
| Share a plan in advance for each session so that the client knows what to expect | 98 |
| Change the environment to reduce sensory demands (e.g., reduce bright lights or distracting noises, provide fiddle toys) | 95 |
| * Provide extra psychoeducation around trauma, arousal and feeling physiologically overwhelmed | 100 |
| Slow down every phase | 98 |
| Use storytelling, perhaps including information from others | 88 |
| Focus on building a positive self-image and coping strategies rather than pathologising and eliminating symptoms | 100 |
| * Prioritise the therapeutic relationship above everything else | 100 |
| * Don’t insist on or encourage eye contact | 95 |
| Use visual or simplified version of ratings scales (e.g., SUDS and VOC) | 81 |
| * Be ready to reformulate throughout the therapy and to shift the focus. Whilst you might start with symptoms, later work could focus on identity, the impact of neurodiversity and adapting to diagnosis | 100 |
| * Keep it simple, even with things that are very complex, and adapt to the person’s level of understanding | 100 |
| Give explicit permission to ask questions | 93 |
| ***Phase 1: History taking*** |  |
| Obtain information from other people as well as the person themselves | 86 |
| Expect to add to history taking throughout the therapy as new information emerges | 100 |
| Focus first on strengths and interests and then move onto problems and history | 93 |
| Vary the way you work (e.g., on the floor, walking, use play, engage with their hobbies and interests) | 86 |
| Create a visual timeline | 88 |
| Spell things out in black and white and be more directive than usual | 95 |
| ***Phase 2: Preparation stage*** |  |
| Use an image of a place rather than imaginal calm place | 98 |
| Think in terms of a ‘positive engaging focus’ rather than necessarily a ‘calm place’ | 95 |
| Be creative with the calm place (e.g., use drawings, emojis, pictures, media clips, animals, fiddle toys, smells) | 93 |
| Encourage them to use stimming behaviour as self-soothing if it works | 81 |
| Use fantasy figures as resources (e.g., superheroes) | 95 |
| Use their special interests and how they feel when engaged in it as a resource | 93 |
| Include exercises to facilitate accessing emotions | 81 |
| Include props to help them identify emotions (e.g., charts about feelings and an emotion wheel) | 81 |
| Install a positive self-view as a resource | 84 |
| Be very clear with clients what this phase is about and why it is necessary | 91 |
| ***Phase 3: Assessment phase*** |  |
| GENERAL |  |
| * Ask for all the elements but if they cannot provide information, go with whatever is given | 100 |
| Assure you are well tuned in to the client before starting this phase | 100 |
| Use a progressive approach to processing, starting with the ‘tip of the finger’ | 80 |
| TARGET |  |
| Use any sensory modality as a target, not necessarily an image | 88 |
| Use a present-day target first | 90 |
| Use a literal description – ask the person to explain what we would see if looking at a photo or a still of a movie. | 88 |
| Proceed without an image if they struggle with finding an image | 95 |
| NEGATIVE COGNITION |  |
| Offer alternatives, prompts and suggestions for cognitions | 98 |
| Skip negative cognition altogether if it causes problems | 93 |
| POSITIVE COGNITION |  |
| Allow the PC to emerge during processing rather than identifying it beforehand | 88 |
| Use more prompts and suggestions to find a PC | 95 |
| Use ‘softeners’ for the PC (e.g., instead of ‘I am strong’ use ‘I am starting to believe that I am strong’) | 93 |
| BODY |  |
| * Be aware of the possibility of sensory overload | 100 |
| ***Phase 4: Desensitisation*** |  |
| Use more directive interweaves than usual | 98 |
| Let the client choose and control length of sets | 98 |
| Use shorter sets and a more frequent return to target | 93 |
| Do not expect generalisation | 90 |
| Repeat their feedback to them to aid processing | 85 |
| Use more physical movement | 80 |
| Start with short sets and build up tolerance from there | 90 |
| ***Phase 5: Installation*** |  |
| Do more cognitive work at this stage if necessary to identify a PC | 85 |
| ***Phase 7: Closure*** |  |
| Take longer to close down and leave extra time for a debrief | 98 |
| End with a relaxing and positive activity | 95 |
| * Offer clear guidance on what to do after the session and what they might experience after the session | 100 |
| Include your own thoughts as part of the debrief | 100 |
| ***Phase 8: Re-evaluation*** |  |
| * Focus on quality of life and functioning to assess progress | 100 |
| Offer your own observations of what has changed | 100 |
| Don’t emphasise keeping logs between sessions if difficult for the client | 95 |

** Re-rated in Round 2*

Table 2 Aspects of EMDR clinical supervision that ≥ 80% of therapists deemed useful when working with autistic clients

|  | % of participants endorsing items 1-3 |
| --- | --- |
| * Peer supervision with time to really discuss cases | 100 |
| Attending an autism SIG | 95 |

** Re-rated in Round 2*

Table 3 Delphi survey statements that failed to attain consensus

|  | % of participants endorsing items 1-2 | % of participants endorsing items 1-3 |
| --- | --- | --- |
| ***General adaptations to EMDR*** |  |  |
| Assess autistic characteristics formally, using self or informant rating scales | 30 | 67 |
| Use CIPOS | 14 | 65 |
| Use ACT strategies | 19 | 70 |
| Use DBT strategies | 19 | 70 |
| Have shorter, more frequent sessions | 12 | 77 |
| Give the option to work remotely | 37 | 70 |
| ***Phase 1: History taking*** |  |  |
| Avoid using clustering | 30 | 79 |
| Use CBT to access core beliefs | 16 | 72 |
| Collect 10 best as well as 10 worst memories | 21 | 63 |
| ***Phase 2: Preparation stage*** |  |  |
| Provide written summaries for the client | 51 | 70 |
| ***Phase 3: Assessment phase*** |  |  |
| GENERAL |  |  |
| Be ready to jump into processing without completing the assessment phase if they are very activated | 16 | 51 |
| Use a child protocol even with adults | 7 | 51 |
| TARGET |  |  |
| Use a physical picture | 10 | 66 |
| POSITIVE COGNITION |  |  |
| Use a visual PC | 7 | 56 |
| Use the opposite of the NC as the PC | 22 | 78 |
| Skip the PC altogether | 7 | 68 |
| VOC |  |  |
| Skip the VOC altogether | 12 | 76 |
| EMOTIONS |  |  |
| Use a visual scale with colours for emotions | 12 | 61 |
| Skip this question if they find it difficult | 15 | 68 |
| SUDS |  |  |
| Skip SUDS if they find this hard, just use their reactions to assess progress | 14 | 76 |
| Make a visual scale with practical examples | 20 | 73 |
| BODY |  |  |
| Use body map to show sensations | 17 | 68 |
| ***Phase 4: Desensitisation*** |  |  |
| Consider an increase in working memory taxation (e.g., using fast eye movements as well as naming colours or spelling) | 13 | 56 |
| Ask about body sensations between each set to keep people in tune with their body | 28 | 75 |
| Try a ‘dry run’ with a non-anxiety provoking memory | 15 | 68 |
| Use the analogy of a remote control, dimmer switch or a cup of water (to measure the SUDS) and draw it so it feels more real | 8 | 45 |
| Use simple proactive interweaves – directive rather than socratic | 38 | 70 |
| Link interweaves to special interests | 33 | 78 |
| ***Phase 5: Installation*** |  |  |
| Use pictures in place of the PC | 8 | 50 |
| Use physical movement to help install a PC | 10 | 43 |
| Skip the installation phase | 0 | 35 |
| Install a neutral rather than positive cognition | 3 | 60 |
| Use visual methods to chart change during installation (e.g., charts, diagrams) | 3 | 58 |
| ***Phase 6: Body scan*** |  |  |
| Use movement and changing position to encourage body awareness | 15 | 60 |
| Walk them through this in more detail (e.g., asking specific questions about each part of the body rather than a general question) | 15 | 70 |
| ***Phase 8: Re-evaluation*** |  |  |
| Obtain information from others about how things have been | 30 | 68 |
| Use a visual chart to emphasise progress | 8 | 70 |
